# Neuroprotective Effects of Baicalein, Wogonin, and Oroxylin A on Amyloid Beta-Induced Toxicity via NF-κB/MAPK Pathway Modulation

**DOI:** 10.3390/molecules25215087

**Published:** 2020-11-02

**Authors:** Yeongseon Ji, Jin Han, Nayoung Lee, Jeong-Hyun Yoon, Kumju Youn, Hyun Joo Ha, Eunju Yoon, Dong Hyun Kim, Mira Jun

**Affiliations:** 1 Department of Food Science and Nutrition, Dong-A University, Busan 49315, Korea; jysun3410@donga.ac.kr (Y.J.); hanj1905@donga.ac.kr (J.H.); kjyoun@dau.ac.kr (K.Y.); hyunjooha0113@gmail.com (H.J.H.); ejyoon@dau.ac.kr (E.Y.); 2 Department of Health Sciences, Dong-A University, Busan 49315, Korea; nylee0420@donga.ac.kr (N.L.); yjhyun1110@donga.ac.kr (J.-H.Y.); mose79@dau.ac.kr (D.H.K.); 3 Department of Medicinal Biotechnology, Dong-A University, Busan 49315, Korea

**Keywords:** Alzheimer’s disease, amyloid beta peptide, *Scutellaria baicalensis*, neuroinflammation, flavones

## Abstract

Amyloid beta (Aβ) peptide, one of the most important pathogenic traits of Alzheimer’s disease (AD), invokes a cascade of oxidative damage and eventually leads to neuronal death. In the present study, baicalein, wogonin, and oroxylin A, main active flavones in *Scutellaria baicalensis*, were evaluated for their neuroprotective effects against Aβ_25–35_-stimulated damage. All tested compounds decreased Aβ_25–35_-induced ROS generation and cell cycle arrest. In particular, baicalein exhibited the strongest antioxidant activity. In addition, these compounds suppressed apoptosis by attenuating mitochondrial dysfunction such as loss of membrane potential, Ca^2+^ accumulation and Bax/Bcl-2 ratio. Furthermore, all tested flavones inhibited the expression of iNOS and COX-2, which resulted in suppressing inflammatory cytokines including TNF-α, NO, and PGE_2_. Noticeably, all compounds exhibited the anti-inflammatory effects through downregulating NF-κB/MAPK pathway. Especially, oroxylin A was effective against both p65 and IκBα, while wogonin and baicalein were suppressed phospho-p65 and phospho-IκBα, respectively. Taken together, baicalein, wogonin, and oroxylin A can effectively relieve Aβ_25–35_-stimulated neuronal apoptosis and inflammation in PC12 cells via downregulating NF-κB/MAPK signaling pathway.

## 1. Introduction

Alzheimer’s disease (AD) is a progressive and age-related neurodegenerative disorder clinically characterized by cognitive decline [1]. It is characterized neuropathologically by the aggregation of amyloid beta (Aβ) peptide in senile plaques and of tau protein in neurofibrillary tangles (NFTs); it is also characterized by pronounced synapse loss, neuronal loss, and gliosis [2]. A recent study suggested that tau can contribute to altering neuronal function in the very early stages of neurodegeneration acting at the presynaptic level [3]. However, many studies have reported that Aβ-induced neurotoxicity including oxidative stress, neuronal apoptosis, and inflammation play a crucial role in AD progression [4,5]. Monomeric Aβ is non-toxic and is easily eliminated from the brain, but aggregated Aβ is neurotoxic and causes malfunction of synaptic receptors and cellular components [6,7,8]. Further, Aβ accumulation may possibly assist tau phosphorylation, neuronal damage, mitochondrial dysfunction, and neuroinflammation. Therefore, identification of promising neuroprotective candidates to prevent and/or eliminate excessive accumulation of Aβ is a prominent strategy for AD treatment.

The mitochondria play an essential role in regulating apoptosis and mitochondrial dysfunction is a hallmark of Aβ-induced toxicity in AD [9]. A previous study reported that Bcl-2 family proteins can induce apoptosis by modulating mitochondrial permeability. Activation of Bax and Bcl-2 family members results in the collapse of mitochondrial membrane potential (MMP), which promotes an overload of intracellular calcium [10,11]. This event leads to the release of pro-apoptotic proteins including cytochrome c or the caspase family into the cytosol. Release of cytochrome c sequentially activates the caspase-9 and -3, which leads to induction of apoptosis.

The NF-κB and MAPK signaling pathways are known to be important in regulating inflammation and are considered to have a crucial function in generating the inflammatory cytokines mediated by Aβ. Under physiological conditions, the heterodimers of NF-κB subunits, mainly p50/p65, are inactivated by association with the inhibitor of κB (IκBα) protein. However, in response to cellular stimuli like Aβ, the IκBα kinase complex undergoes phosphorylation, leading to NF-κB nuclear translocation, and its binding to the specific promoters of genes encoding pro-inflammatory enzymes including cyclooxygenase-2 (COX-2) and inducible nitric oxide synthase (iNOS). The MAPK signaling pathway including p38, JNK, and ERK kinase, is known to be promoted by inflammatory stimuli and controls NF-κB activation to promote inflammation [12].

*Scutellaria baicalensis (S. baicalensis)* has been commonly used as a herbal tea in Asia, Europe, and North America [13]. Baicalein, wogonin, and oroxylin A, the major bioactive flavones in *S. baicalensis*, have a wide range of biological properties such as antioxidant, anti-inflammatory, and hepatoprotective effects [14]. Moreover, recent studies demonstrated that the compounds exerted novel potentials in neuroprotection. Baicalein exhibited marked improvement of cognitive deficits induced by chronic cerebral hypoperfusion through reducing levels of oxidative stress markers [15]. Oroxylin A reduced cognitive impairment caused by permanent occlusion of bilateral common carotid arteries (2VO) via inhibiting activated microglia and enhancing expression of brain derived neurotrophic factor (BDNF) and cAMP response element-binding protein (CREB) [16]. In the case of wogonin, the compound recovered brain injury against ischemic stroke or gamma irradiation by anti-inflammatory activity [17,18]. In addition, our previous study exhibited that baicalein prevented the production of Aβ and senile plaque through inhibiting BACE1 and AChE [19]. However, the inhibitory activity of these flavones against Aβ-evoked neurotoxicity are relatively unknown. Therefore, our study was designed to explore the effects and underlying mechanism of three compounds against Aβ-mediated neuronal inflammation and apoptosis in PC12 cells.

## 2. Results and Discussion

### 2.1. Effect of Baicalein, Wogonin and Oroxylin A on Aβ_25–35_-Induced Cytotoxicity in PC12 Cells

**Figure 1:**
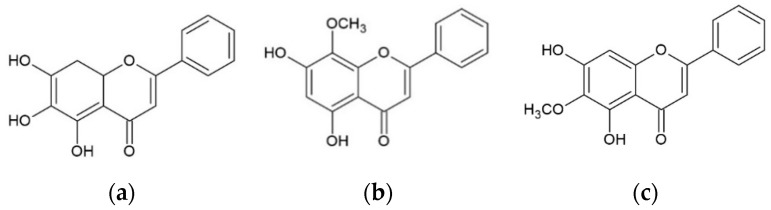
The chemical structures of (**a**) baicalein, (**b**) wogonin, and (**c**) oroxylin A.

Baicalein, wogonin, and oroxylin A share very similar chemical structures (Figure 1). The differences in the chemical structures of the three flavones are the number and position of hydroxyl and methoxy groups. The only difference between baicalein and oroxylin A is at the C-6 position, in which the hydroxyl group at C-6 of baicalein was methylated in oroxylin A. The difference between oroxylin A and wogonin is the location of the methoxy (-OCH_3_) group with the -OCH_3_ group of oroxylin A at the C-6 position while that of wogonin is at the C-8 position.

**Figure 2:**
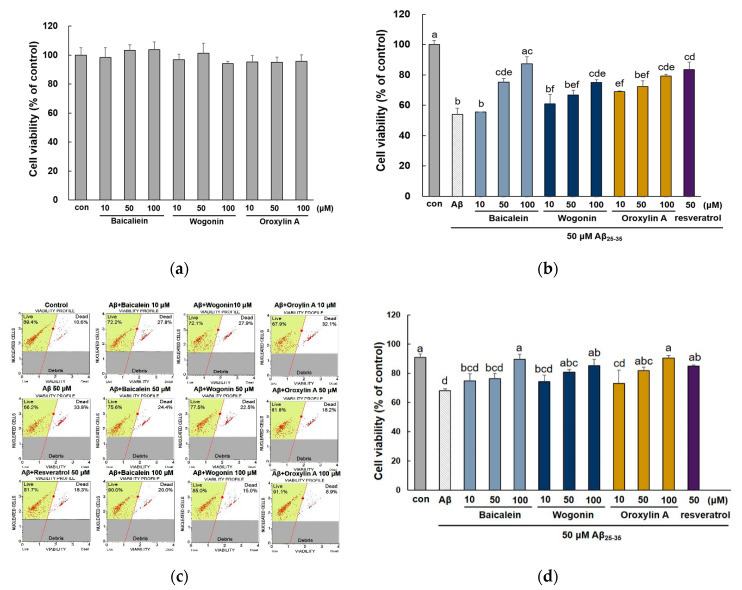
Protective effects of baicalein, wogonin, and oroxylin A in Aβ_25–35_-treated PC12 cells. Measurement of cytotoxicity due to baicalein, wogonin, and oroxylin A in PC12 cells (**a**). PC12 cells were treated with three compounds for 1 h followed by exposure to 50 μM Aβ_25–35_ for 24 h, and cell viability was determined by MTT assay, (**b**) and flow cytometry (**c**). Statistical analysis results of the percentage of cell viability by flow cytometry (**d**). Different alphabets indicated significant difference at *p* < 0.05.

To evaluate the cytotoxic effect of the three compounds on Aβ_25–35_-induced PC12 cells, we performed a cell viability assay. As shown in Figure 2a, the three compounds did not significantly affect the viability of PC12 cells up to 100 μM. As a result of Aβ_25–35_-induced injury, the cell viability was decreased to 54.01 ± 4.13% of the controls (Figure 2b). When cells were pretreated with baicalein, wogonin, or oroxylin A, cell viability was improved in a dose-dependent manner. In particular, 10 μM oroxylin A showed remarkable recovery against Aβ_25–35_-stimulated cell viability (68.95 ± 0.50%), higher than baicalein and wogonin used at the same concentration (55.59 ± 0.07% and 60.92 ± 6.27%, respectively). In contrast, baicalein showed a somewhat weaker effect at 10 μM, but substantially restored Aβ_25–35_-induced cytotoxicity at 50 and 100 μM (75.36 ± 2.39% and 87.41 ± 4.59%, respectively).

Cell viability was also evaluated using double DNA intercalating fluorescent dyes (Figure 2c,d). Aβ_25–35_ exposure reduced cell viability to 74.77 ± 1.60% compared with the control group. In contrast, the three flavones significantly attenuated the reduced cell viability (*p* < 0.05) and all tested flavones markedly improved cell viability close to the control group (98.60 ± 3.67, 93.62 ± 4.98, and 99.56 ± 1.87%, respectively).

**Figure 3:**
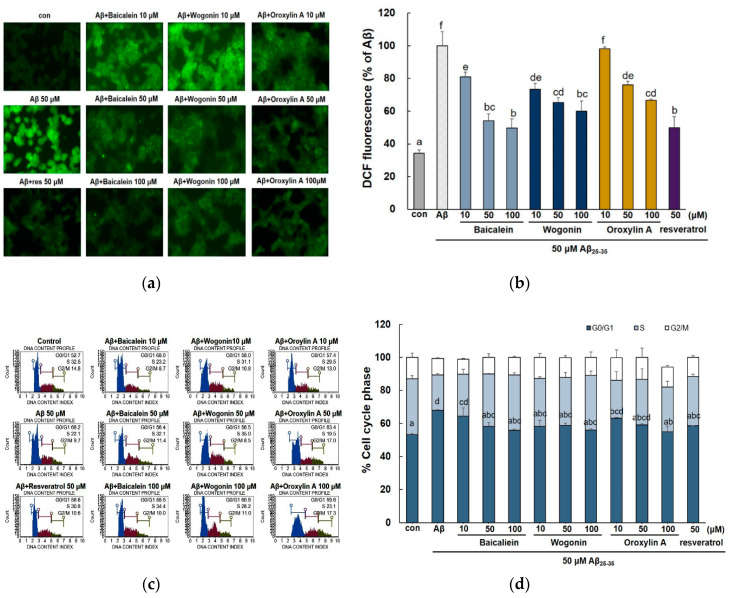
Effects of baicalein, wogonin and oroxylin A on intracellular ROS levels and cell cycle arrest in Aβ_25–35_-induced PC12 cells. The cells were pretreated with samples for 1 h, and stimulated with Aβ_25–35_ for 24 h. ROS levels were observed using CM-H_2_DCFDA (**a**) and measured by microplate reader (**b**). Cell cycle was measured by flow cytometry (**c**). Statistical analysis results of the percentage of cell cycle by flow cytometry (**d**). Different alphabets indicated significant difference at *p* < 0.05.

Previous studies have shown that Aβ can induce the formation of ROS, which lead to an increase in oxidative stress, and excessive ROS levels may activate neuronal cell death in patients with AD [20]. In addition, the presence of transition metals including copper (Cu), zinc (Zn), and iron (Fe) within the senile plaques in AD patients indicated that the transition metals may directly interact with Aβ and induce severe oxidative stress condition [21]. Therefore, intracellular ROS levels were measured to indicate the Aβ-stimulated oxidative stress. As shown in Figure 3a,b, Aβ_25–35_ treatment considerably increased the production of ROS by about three-fold compared with the control group. However, pretreatment of PC12 cells with the three compounds significantly eliminated ROS accumulation (*p* < 0.05). Baicalein and wogonin strongly attenuated oxidative stress even at the lowest concentration (10 μM), which was shown to be significant (*p* < 0.05). In particular, 50 μM baicalein had a strong inhibitory effect (54.23 ± 4.21%) similar to that of resveratrol, the positive control (49.84 ± 6.69%) at the same dose.

Generally, antioxidant activity depends on the position and number of the hydroxyl groups and other substituents. The structural criteria for the antioxidative activity of flavonoids include the catechol group in the A-ring or in the B-ring [22]. As shown in Figure 1, baicalein has catechol moieties in the A-ring, which provide antioxidant properties. With a catechol group in the A-ring, baicalein is a better antioxidant than wogonin and oroxylin A with no catechol group, according to our results. Furthermore, baicalein also possesses more hydroxyl groups in the A-ring compared to wogonin and oroxylin A, resulting in higher electron transfer or hydrogen donating ability. On the contrary, for wogonin and oroxylin A, the presence of methoxy groups at the A-ring increased the steric hindrance, which is adverse to the electron transfer process, which may partially contribute to the low antioxidant activity. In agreement with these findings, previous studies demonstrated that baicalein possesses a higher antioxidant effect than wogonin and oroxylin A against lipid-peroxidation in lung tissues and radical scavenging activity [23,24]. In another study, Cai et al. showed that baicalein has high radical scavenging activity, whereas other flavones like chrysin (5,7-dihydroxyflavone) exhibited far lower such activity [22]. Furthermore, baicalein strongly suppressed the Fe-induced Fenton chemistry via chelation and radical scavenging mechanism [25].

To determine whether the cytotoxic effect of Aβ on PC12 cells was caused by cell cycle arrest, the effect of *S. baicalensis* on cell cycle distribution in Aβ-stimulated PC12 cells was measured by flow cytometry. As shown in Figure 3c,d, Aβ_25–35_ markedly increased the proportion of cells in the G0/G1 phase to 67.9 ± 0.42%, indicating that Aβ treatment induced G1 phase arrest. However, all three compounds inhibited cell cycle arrest. In a previous study, baicalein was revealed to induce tumor cell cycle arrest, and wogonin induced G1 phase arrest in human colorectal cancer cells [26,27]. Therefore, our results suggest that baicalein, wogonin, and oroxylin A could regulate cell cycle progression in Aβ_25–35_-stimulated PC12 cells.

### 2.2. Effect of Three Flavones on Mitochondria Dependent Apoptosis in PC12 Cells

**Figure 4:**
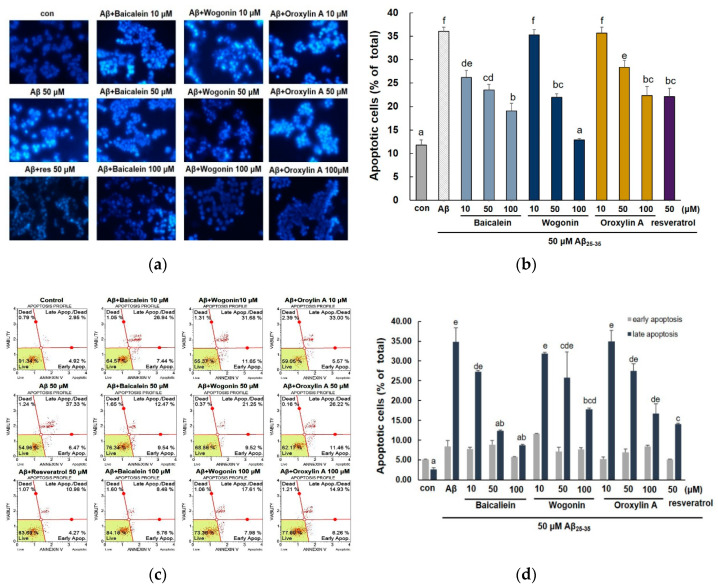
Effects of baicalein, wogonin, and oroxylin A on Aβ_25–35_-stimulated apoptosis in PC12 cells. The cells were pretreated with samples for 1 h, and stimulated with Aβ_25–35_ for 24 h. Apoptotic cells were stained with Hoechst 33,342 (**a**). Quantification of apoptotic cells (**b**). Early and late apoptosis measured by flow cytometry (**c**). Statistical analysis results of the percentage of apoptosis by flow cytometry (**d**). Different alphabets indicated significant difference at *p* < 0.05.

Several studies have shown that Aβ toxicity increases neuronal cell death and it exhibit the characteristics of apoptosis [28]. In addition, it has been reported that Aβ-induced oxidative stress leads to apoptotic neuronal cell death by formation of ROS [29]. As shown in Figure 4a, the nuclei of control cells showed rounded shape and no condensation, indicating that there were no apoptotic cells. After 24-h exposure to Aβ_25–35_, the cells showed crescent shaped nuclei and fragmentation with bright blue fluorescence, suggesting that these cells underwent severe morphological change indicative of apoptosis. As presented in the histogram of Hoechst 33,342 staining results (Figure 4b), Aβ_25–35_ treatment increased the cellular apoptotic rate to 36.05 ± 0.89%, while pretreatment with three compounds apparently decreased apoptotic cells. In particular, baicalein showed potent activity even at 10 μM (26.22 ± 1.49%), and 100 μM wogonin attenuated apoptosis (12.8 ± 0.33%) close to the control level (11.75 ± 1.16%).

To confirm the inhibitory property of three flavones against Aβ_25–35_-induced PC12 cells, Annexin V/PI staining was used to analyze early and late stage apoptotic cells. As shown in Figure 4c, after the treatment of the cells with 50 μM Aβ_25–35_, the late apoptosis was increased to 34.80 ± 3.58 %. After the pretreatment of the cells with three flavones, the late apoptotic cells were decreased, in particular, baicalein markedly attenuated late apoptosis ratio at 50 and 100 μM (12.28 ± 0.27% and 8.66 ± 0.25%, respectively).

**Figure 5:**
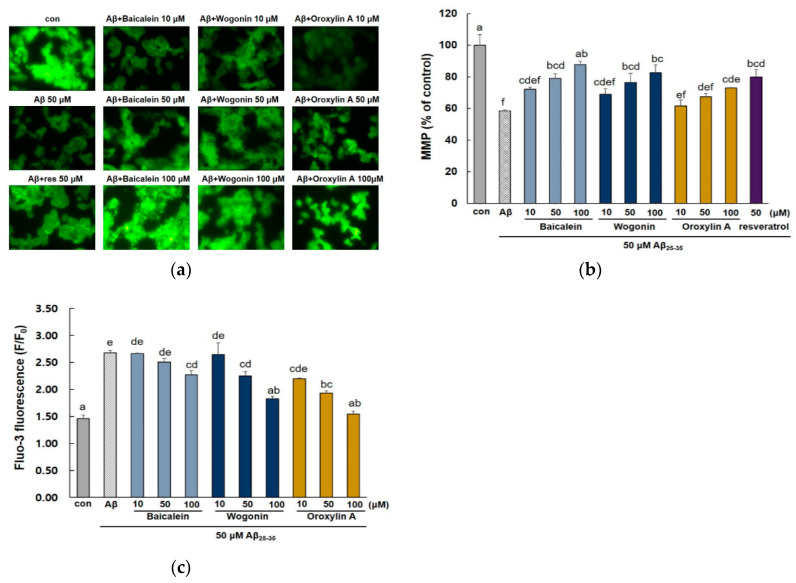
Effects of baicalein, wogonin, and oroxylin A on Aβ_25–35_ induced mitochondrial dysfunction. The cells were pretreated with samples for 1 h and stimulated with Aβ_25–35_ for 24 h. Mitochondrial membrane potential (MMP) was determined by Rhodamine 123 staining (**a**). Statistical analysis results of the percentage MMP through Rhodamine 123 intensity (**b**). Intracellular Ca^2+^ levels were analyzed using Fluo-3AM (**c**). Different alphabets indicated significant difference at *p* < 0.05.

Aβ_25–35_-induced apoptosis can be activated by oxidative stress via mitochondrial dysfunction, which leads to the collapse of mitochondrial membrane potential (MMP) and an increase in intracellular Ca^2+^ levels [30]. In this study, Aβ_25–35_ exposure dramatically reduced the fluorescence intensity (58.48 ± 0.66%) compared with the control group, indicating that reduced mitochondrial membrane potential (Figure 5a,b). In contrast, the three compounds showed significantly restored MMP (*p* < 0.05), indicating that baicalein, wogonin, and oroxylin A inhibit Aβ_25–35_-induced MMP collapse in PC12 cells. In addition, 50 μM baicalein and wogonin showed potent activity in restoring MMP to similar levels (79.02 ± 2.88% and 76.33 ± 5.76%, respectively) as the positive control (79.96 ± 4.74%). As shown in Figure 5c, after the treatment of cells with 50 μM Aβ_25–35_, the intracellular calcium level [Ca^2+^]_i_ was increased by about two-fold compared with the control group. On the contrary, preincubation of cells with the three flavones resulted in a significant decrease in the elevated [Ca^2+^]_i_.

**Figure 6:**
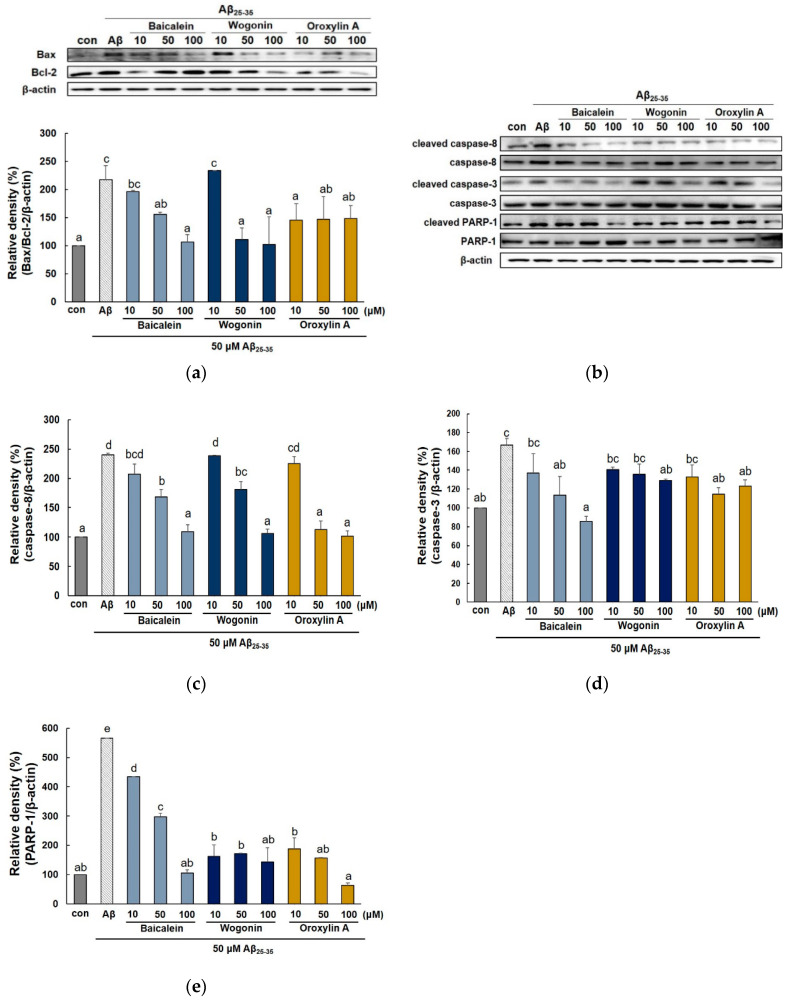
Effects of baicalein, wogonin, and oroxylin A on the expression of apoptosis-related biomarkers in Aβ_25–35_-induced PC12 cells. The cells were pretreated with samples for 1 h and stimulated with Aβ_25–35_ for 24 h. Protein expression of Bax/Bcl-2 ratio, caspase-8, caspase-3, and PARP1 were determined using a western blotting (**a**–**e**). Different alphabets indicated significant difference at *p* < 0.05.

Expression of Bcl-2 family and caspase family proteins has been well known to regulate the apoptotic pathway in mitochondria. As shown in Figure 6a, pre-treatment with the three flavones significantly suppressed the upregulation of Bax expression and downregulation of Bcl-2 expression induced by Aβ_25–35_. Oroxylin A did not affect Bcl-2, but markedly decreased Bax even at 10 μM. On the contrary, baicalein and wogonin showed a weaker inhibitory effect on Bax expression compared to oroxylin A, but effectively increased Bcl-2 at 100 μM.

To determine whether the extrinsic or the intrinsic apoptosis pathway is related to Aβ toxicity, the expression of caspase-8 (extrinsic), caspase-3, and PARP-1 (intrinsic) were measured. As shown in Figure 6b, the level of caspase family proteins and PARP-1 in PC12 cells was increased by the Aβ_25–35_ treatment. However, all three compounds suppressed the expression of apoptosis-related proteins in dose-dependent manner. In particular, 50 μM oroxylin A decreased the expression of cleaved caspase-8 and PARP-1 to a level similar to the control (113 ± 14.64% and 156.17 ± 28.52%, respectively) (Figure 6c,e). Moreover, among the three compounds, baicalein showed the highest inhibitory activity against cleaved-caspase-3 (Figure 6d). Overall, these results suggest that baicalein, wogonin, and oroxylin A effectively suppressed caspase-8, -3, and PARP-1, indicating that the three flavones of *S. baicalensis* inhibit apoptosis via both extrinsic and intrinsic pathways.

### 2.3. Effect of Three Flavones on Inflammatory Cytokines in Aβ_25–35_-Stimulated PC12 Cells

**Figure 7:**
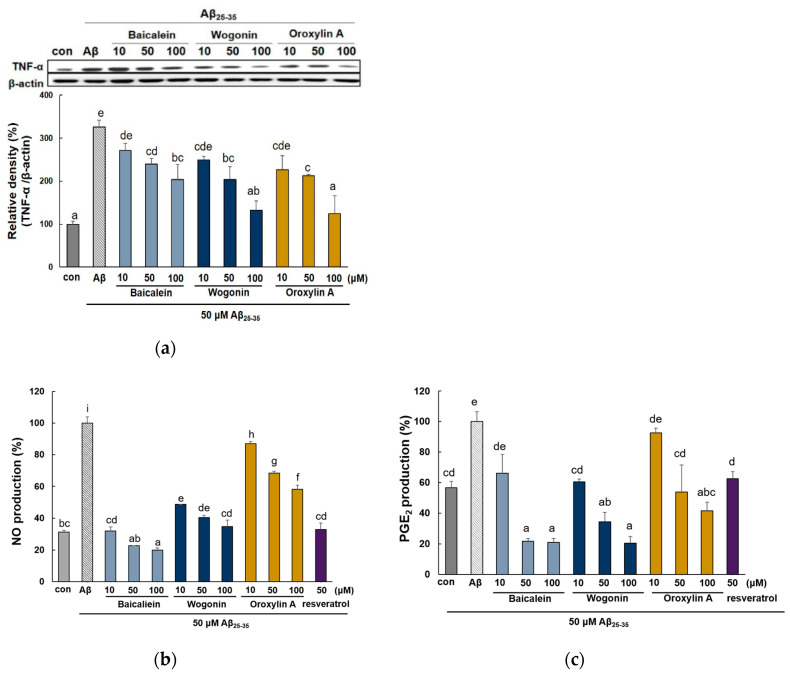

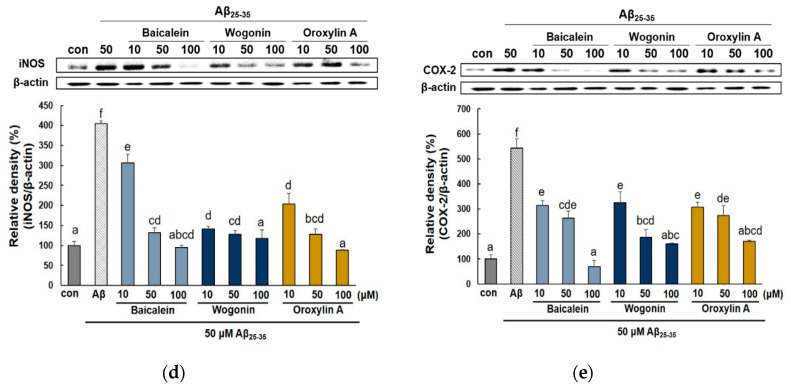
Effect of baicalein, wogonin, and oroxylin A on the production of proinflammatory cytokines and their protein expressions in Aβ_25–35_-induced PC12 cells. The cells were pretreated with samples for 1 h, and stimulated with Aβ_25–35_ for 24 h. The media were collected to assess the production of no and PGE_2_. The expression of TNF-α (**a**), iNOS (**d**), and COX-2 (**e**). NO levels were assessed by Griess assay (**b**). PGE_2_ levels in supernatants were determined by ELISA assay kit (**c**). The PC12 cells were incubated with samples and then treated with Aβ_25–35_ for 24 h. The cells were lysed and the protein levels were quantified using the BCA assay. Different alphabets indicated significant difference at *p* < 0.05.

Aβ can directly activate inflammatory responses in the brain of AD patients, followed by the production of excessive proinflammatory and neurotoxic factors, including the cytokine TNF-α, NO, and PGE_2_ [31]. Therefore, we investigated the inhibitory effects of baicalein, wogonin, and oroxylin A against inflammatory cytokines in Aβ_25–35_-induced PC12 cells. Aβ_25–35_ treatment for 24 h increased TNF-α expression by more than three-fold compared to the control (Figure 7a). On the contrary, treatment with the three flavones inhibited the TNF-α level. Wogonin and oroxylin A at 100 μM reduced the expression of TNF-α to the control levels, whereas baicalein displayed a somewhat weaker effect.

Aβ_25–35_ stimulation resulted in an increased level of NO and PGE_2_ production (Figure 7b,c). All the tested compounds significantly attenuated the expression of NO_._ 50 and 100 μM of baicalein remarkably inhibited these to levels lower (22.72 ± 0.01 and 19.93 ± 1.32%, respectively) than that of the control group (31.41 ± 1.08%). Pretreatment of baicalein, wogonin, and oroxylin A inhibited the Aβ_25–35_-induced PGE_2_ production. Baicalein and wogonin showed the most notable ability to inhibit PGE_2_ levels.

As shown in Figure 7d,e, Aβ_25–35_ markedly induced the expression of iNOS and COX-2 protein by more than four-fold versus the control group. However, all the tested compounds obviously suppressed the level of both iNOS and COX-2. Interestingly, wogonin and oroxylin A showed a stronger inhibitory activity against iNOS than baicalein at 10 μM. In case of the inhibitory effect against COX-2, baicalein suppressed the protein expression of COX-2 to the control levels.

### 2.4. Effect of Three Flavones on NF-κB and MAPK Pathway

**Figure 8:**
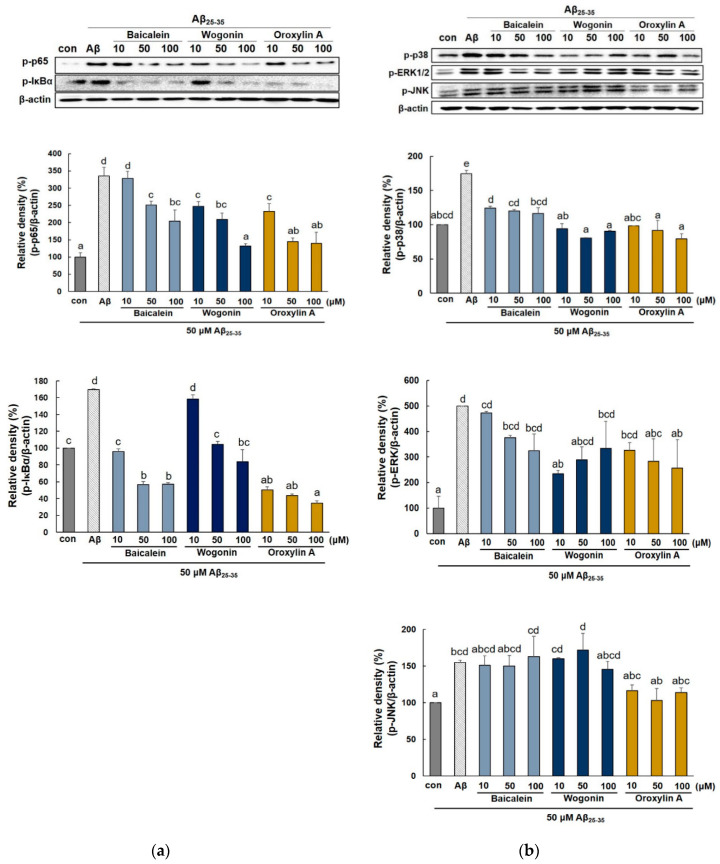
Effects of baicalein, wogonin, and oroxylin A on NF-κB and MAPK expression in Aβ_25–35_-induced PC12 cells. Expression of p-p65, p-IκB (**a**) and MAPKs (**b**). Cells were pretreated with the compounds for 1 h, and then incubated with Aβ_25–35_ for 4 h. The cells were lysed and the protein levels were quantified using the BCA assay. Different alphabets indicate significant difference at *p* < 0.05.

NF-κB is crucial in the regulation of proinflammatory mediators such as iNOS and COX-2. To further explore the mechanism of baicalein, wogonin, and oroxylin A against neuronal inflammation of Aβ_25–35_-stimulated PC12 cells, the expression of NF-κB and p-IκBα protein were detected. As shown in Figure 8a, phospho-p65 (NF-κB subunit) was notably induced in Aβ_25–35_-stimulated PC12 cells, and this effect was blocked by treatment with the three flavones. Especially, oroxylin A showed a stronger inhibitory effect against both phosphor-p65 and phospho-IκBα while wogonin and baicalein were suppressed phospho-p65 and phospho-IκBα, respectively.

Activation of the NF-κB signaling pathway is intimately involved with MAPK activation [12]. Thus, we investigated the effects of flavones against MAPKs in Aβ_25–35_-stimulated PC12 cells. As shown in Figure 8b, the expression of p38, ERK, and JNK MAPK were obviously increased in Aβ_25–35_-induced PC12 cells (174.3 ± 5.05%, 501.58 ± 0.70%, and 155.35 ± 2.55%, respectively). However, all three flavones blocked the phosphorylation of p38. Wogonin remarkably inhibited the phosphorylation of ERK, but at 50 and 100 μM, it showed weaker activity (289.57 ± 51.37% and 394.14 ± 23.38%) compared to 10 μM wogonin (235.86 ± 13.26%). In case of JNK, oroxylin A effectively suppressed the phosphorylation of JNK at all concentrations (116.61 ± 7.72%, 103.145 ± 16.46%, and 113.91 ± 6.44%, respectively).

Previous studies have indicated that baicalein and wogonin can attenuate the LPS-induced inflammatory response by downregulating the NF-κB and MAPK signaling pathways [32,33]. There are limited studies on the effects of oroxylin A on the MAPK pathway, and the present study newly revealed that the compound effectively downregulated the MAPK family.

Although these flavones have neuroprotective effects, additional studies are needed to determine whether the flavones of *S. baicalensis* can pass through the blood-brain barrier (BBB) after administration. Furthermore, the use of many natural products as neuroprotective agents has been hampered by BBB impermeability. In an earlier study, Tsai et al. revealed that baicalein could penetrate the BBB in 20–30 min after administration [34]. In another study, the presence of wogonin has been shown in the mouse brain after intravenous administration at 20 mg/kg [35]. In addition, Fong et al. found that oroxylin A could also cross the BBB, with brain concentrations ranging from 7.9 to 224 pmol/g, which is the highest concentration among that reported for the three flavones [36].

For thousands of years, *S. baicalensis* has been widely regarded as a safe and non-toxic traditional medicine. Clinically, Li et al. have demonstrated that single oral doses of 100 mg to 2800 mg of baicalein are safe [37]. Furthermore, sub-chronic toxicity studies in beagle dogs have demonstrated that wogonin had no organ toxicity after chronic intravenous administration at dosages of 60 mg/kg [38]. However, there has been no research about the safety or clinical data of oroxylin A till date. Li et al. showed that orally, 80 mg/kg of oroxylin A combined with 200 mg/kg imatinib, inhibited tumor growth in mice, whereas there were no significant changes in the body weight, heart, liver, spleen, and kidney [39]. These results showed the safety of the three flavones but more additional studies on the toxicity of oroxylin A are needed. This section may be divided by subheadings. It should provide a concise and precise description of the experimental results, their interpretation as well as the experimental conclusions that can be drawn.

## 3. Materials and Methods

### 3.1. Reagents

Wogonin (purity ≥ 98%) and oroxylin A (purity ≥ 98%) were obtained from Chemfaces (Wuhan, China). PC12 cells were obtained from American Type Culture Collection (ATCC). Roswell Park Memorial Institute (RPMI) cell culture medium, phosphate buffered saline (PBS), fetal bovine serum (FBS), trypsin-EDTA, donor equine serum, and penicillin solution were purchased from Hyclone Laboratories (Logan, UT, USA). RPMI 1640 phenol red free medium and HBSS were supplied from Gibco BRL (Grand Island, NY, USA). N_2_ supplement, CM-H_2_DCFDA, Hoechst 33342, fluo-3/AM and pluronic F-127 were supplied from Invitrogen (Carlsbad, CA, USA). Aβ_25–35_ (purity ≥ 97%), baicalein (purity ≥ 98%), resveratrol, MTT reagent, Rhodamine123, and were purchased from Sigma-Aldrich (St. Louis, MO, USA). The specific antibodies for Bax, Bcl-2, caspase-8, -3, PARP-1, iNOS, COX-2, TNF-α, β-actin and horseradish peroxidase-conjugated secondary antibodies were obtained from Santa Cruz Biotechnology Inc. (Santa Cruz, CA, USA). The antibodies against phospho-IκB-α, phospho-p65, phospho-p38, phospho-JNK, phospho-ERK1/2 antibodies were purchased from Cell Signaling Technology Inc. (Beverly, MA, USA). AnnexinV and Dead Cell, Cell Cycle, and Count and Viability Kit were obtained from Merck Millipore (Darmstadt, Germany). PGE_2_ enzyme immunoassay kit (Parameter™) was obtained from R&D System (Minneapolis, MN, USA). All other chemicals used were of analytical grade commonly available.

### 3.2. Aggregation of Aβ and Cell Culture

Aβ_25–35_ was solubilized in DMSO (Sigma-Aldrich) and diluted with PBS. The stock solution allowed to aggregate by incubating at 37 °C for 48 h. PC12 cells were cultured in RPMI 1640 at 37 °C in an atmosphere of 5% CO_2_. PC12 cells were plated 6- or 96-well plate, and treated with 10, 50, or 100 μM tested compounds for 1 h before treatment of 50 μM Aβ_25–35_.

### 3.3. Determination of Cell Viability

PC12 cells were seeded in a 96-well plate at 5 × 10^4^ cells per well and pretreated with tested compounds for 1 h and then exposed to Aβ_25–35_ for 24 h. Then, 10 μL/well of MTT solution was added and cells were incubated at 37 °C for 3 h. The supernatants were then removed, and formazan crystals were dissolved in DMSO. The amount of soluble formazan was measured at 570 nm (ELX808, Winooski, VT, USA). Cell viability was also measured by flow cytometry Muse™ Count & Viability kit and using Muse™ Cell Analyzer (Millipore, Billerica, MA, USA).

### 3.4. Detection of Intracellular Reactive Oxygen Species (ROS)

ROS production was quantified by using 2′,7′-dichlorodihydrofluoresce in diacetate (DCF-DA) dye. PC12 cells were cultured on 96-well plates as described and incubated with 10 μM DCF-DA for 30 min at 37 °C and then washed with HBSS. The fluorescent intensity of ROS was measured by a fluorescence at excitation and emission wavelengths of 485 and 528 nm, respectively (FLX800, Winooski, VT, USA). For ROS imaging, fluorescence microscopy was employed (×400, Olympus, Tokyo, Japan).

### 3.5. Measurement of Apoptosis

After the 24 h treatments, cells were fixed with 4% formaldehyde and incubated with Hoechst 33,342 solution at 37 °C for 15 min. A fluorescent microscope was used to observe morphology change of apoptotic cells. Apoptotic cell was also determined by flow cytometry with the Muse™ Annexin V and Dead Cell kit according to the manufacturer’s instruction.

### 3.6. Cell Cycle Analysis

PC12 cells were seeded into 48-well plates at a density of 5 × 10^5^ per well. Adherent cells were collected with trypsin-EDTA and fixed in 70% ethanol for 3 h at 20 °C. The cells were washed with PBS and incubated with cell cycle reagent for 30 min at room temperature in the dark. Then, the results were analyzed using Muse™ Cell Analyzer.

### 3.7. Assessment of MMP and Intracellular Calcium Level

Cultured PC12 cells were stained with Rhodamine 123 and incubated at 37 °C for 30 min. The cells were washed and observed using fluorescence microscopy. The fluorescent intensity of MMP was measured by a fluorescence at excitation and emission wavelengths of 485/528 nm.

### 3.8. Evaluation of NO and PGE_2_ Production

To evaluate the inhibitory effect of tested compounds on NO, Griess assay was performed. Equal volume of media and Griess reagent were mixed and incubated for 10 min at room temperature. The absorbance was detected by a microplate reader at 570 nm. The NO concentration was calculated using a standard curve from NaNO_2_.

The PGE_2_ generation in the supernatant was determined by using a PGE_2_ enzyme immunoassay kit according to the guidelines furnished by the manufacturer’s instruction.

### 3.9. Western Blot Analysis

Pretreated PC12 cells were rinsed with cold PBS and suspended in a lysis buffer on ice for 1 h. The cell lysate was then centrifuged at 13,000 rpm for 10 min at 4 °C. Protein quantification was determined using the BCA assay. Equal concentration of proteins were electrophoresed in a SDS-PAGE and then transferred onto PVDF membranes. The membranes were blocked in 5% skim milk at room temperature for 2 h, and incubated overnight in the primary antibody solution (β-actin, Bcl-2, Bax, caspase-8, PARP-1, TNF-α, iNOS, caspase-3, p-IκB-α, p-p65, p-p38, p-ERK1/2 and p-JNK) at 4 °C. Then the membranes were probed with appropriate secondary antibodies for 1 h at 37 °C. After washing with PBST, detection was carried out using Atto EZ-capture (Tokyo, Japan).

### 3.10. Statistical Analysis

Data were expressed as mean ± SD, and each experiment was repeated three times. Statistical analyses were performed using SAS software (version 9.3, SAS Institute, Cary, NC, USA). One-way analysis of variance (ANOVA) with post hoc Tukey test were used to assess for the multiple comparisons. Different alphabet letters were considered significant when *p* < 0.05.

## 4. Conclusions

In summary, our experiments show that the baicalein, wogonin, and oroxylin A exhibited neuroprotection of PC12 cells against Aβ_25–35_ through multiple mechanisms including restriction of oxidative stress, mitochondria-mediated apoptosis, and neuroinflammation. Especially, baicalein showed the highest activity in oxidative stress whereas wogonin had an excellent inhibitory activity on apoptosis. Moreover, all the tested flavones attenuated Aβ_25–35_-induced mitochondrial dysfunction; in particular, baicalein showed potent activity in restoring MMP whereas oroxylin A exhibited strong inhibitory activity on Ca^2+^ accumulation. In the apoptotic pathway, all tested flavones suppressed both the intrinsic and extrinsic apoptotic pathways through inhibition of cleaved caspase-8, -3, and PARP-1. Further, the three flavones induced inflammatory cytokines and mediators such as TNF-α, NO, PGE_2_, iNOS, and COX-2 via regulation of the NF-κB/MAPK pathway.

Collectively, all three compounds attenuated Aβ_25–35_-induced neurotoxicity but induced different neuroprotective mechanisms. Thus, our results suggest that baicalein, wogonin, and oroxylin A might provide neuroprotection through synergistic interaction.
